# Fortunellin Elevates the AMPK Pathway to Reduce Lipid Deposition and Immune Disorders in Young Non-Alcoholic Fatty Liver Disease Rats

**DOI:** 10.2174/0118715303391165250828111133

**Published:** 2025-09-01

**Authors:** Nan Ping, Na Qin

**Affiliations:** 1 Department of Pediatrics, Heping Hospital Affiliated to Changzhi Medical College, Changzhi City,046000, Shanxi Province, China

**Keywords:** Fortunellin, NAFLD, AMPK, lipid deposition, immune disorders, liver injury

## Abstract

**Introduction:**

Non-alcoholic fatty liver disease (NAFLD) is the leading cause of chronic liver disease and seriously threatens children’s health. Fortunellin exerts a protective role in several human diseases, but its function in NAFLD is unclear. This research tried to uncover Fortunellin’s function and mechanism in young NAFLD rats.

**Methods:**

A young rat model of NAFLD was established by administering a high-fat diet (HFD). Also, Fortunellin was delivered *via* intragastric administration. The effects of Fortunellin on NAFLD were assessed through hematoxylin-eosin staining, analysis of serum levels of ALT, AST, TCH, TG, LDL-C, and HDL-C, Oil Red O staining, Western blot, ELISA, and quantitative real-time PCR (qRT-PCR). Additionally, the Fortunellin mechanism in NAFLD was estimated with Western blot, immunofluorescence, Oil Red O staining, and ELISA assays.

**Results:**

Functionally, Fortunellin (5 or 10 mg/kg) reduced liver injury in young NAFLD rats, which was mainly associated with the gradual decrease of a liver index, the increased liver tissue score, and the gradually decreased serum levels of ALT and AST. Also, Fortunellin restrained NFALD rat dyslipidemia by lessening TCH, TG, LDL-C serum levels, and increasing HDL-C levels. Furthermore, Fortunellin repressed liver lipid metabolism and immune disorders in young NAFLD rats. Mechanically, Fortunellin enhanced the AMPK activation in young NAFLD rats. Additionally, Fortunellin relieved lipid deposition and immune disorders in young NAFLD rats, while compound C (CC, an AMPK inhibitor) abolished these impacts.

**Discussion:**

This study confirmed that Fortunellin alleviated liver injury in young rats with NAFLD, and this might be achieved by activating the AMPK axis. The completion of this study provided a promising drug for the NAFLD treatment.

**Conclusion:**

In summary, Fortunellin alleviated lipid deposition and immune disorders in young rats with NAFLD through the activation of AMPK.

## 1 INTRODUCTION

Non-alcoholic fatty liver disease (NAFLD) is a progressive liver disease frequently observed in children [1, 2]. The progression of NAFLD typically advances from simple steatosis to non-alcoholic steatohepatitis, cirrhosis, and ultimately hepatocellular carcinoma [3]. The onset of NAFLD is often associated with excessive dietary calories, diseases,and drugs [4]. According to incomplete statistics, NAFLD is a significant cause of chronic liver disease in children, affecting nearly 10% of the global pediatric population [5]. However, the molecular pathogenesis of NAFLD in children has remained largely elusive. Thus, the therapy options for NAFLD in children remain limited.

An increasing body of research indicates that plant extracts possess a protective function in various human diseases including NAFLD [6, 7]. Fortunalin is a flavonoid collected from *Fortunella margarita* (kumquat) fruit [8]. As previously reported, Kumquat extract exerts anti-tumor, antioxidant, and anti-inflammatory effects, thereby playing a protective role in human health [9]. Fortunellin shares similar functions with kumquat extract and is recognized as a valuable anti-inflammatory agent. Such as, Fortunellin improves inflammation in sepsis-induced acute kidney injury (AKI) through TLR4/NF-κB, hinting that Fortunellin possesses potential as a therapeutic drug for sepsis-induced AKI [10]. Fortunellin protects against fructose-induced oxidative stress *via* AMPK/Nrf2 in fructose-treated diabetic mice [8]. Crucially, Fortunellin has been proven to be non-toxic because it does not interact with CYPs involved in drug metabolism, further indicating that Fortunellin is considered promising in early drug research [11]. Nevertheless, the Fortunellin function in NAFLD in children has not been illustrated. Accordingly, we tried to elucidate the potential role and mechanism of Fortunellin in young rats with NAFLD.

The innovation of this study lies in that we explored the potential role and mechanism of Fortunellin in young rats with NAFLD for the first time. In the current study, we built a young rat NAFLD model *via* feeding a high-fat diet (HFD, 69% basic feed, 10% lard oil, 2% cholesterol, 5% sugar, 0.5% cholate, 10% yolk powder, 3% yeast powder, and 0.5% decavitamin). Our data preliminarily corroborated that Fortunellin lessened body weight and liver weight index in young NAFLD rats. These findings suggested that Fortunellin might serve as an effective agent for alleviating NAFLD in children. Subsequently, we further revealed the other functions and potential molecular mechanisms of Fortunellin in NAFLD in children.

## 2 MATERIALS AND METHODS

### 2.1 Construction of Rat NFALD Model

Male young Sprague-Dawley (SD) rats (3-week-old, 50-70g) were purchased from Cyagen (Suzhou, China). The rats were acclimatized for one week in a controlled environment with a 12-hour sleep/wake cycle at a temperature of 22±1°C. Animal assays were approved by the Ethics Committee of Heping Hospital Affiliated to Changzhi Medical College (Changzhi City, Shanxi Province, China, 2023012) and followed the institutional/national/ international guidelines for the care and use of laboratory animals.

All rats were randomly divided into control (n=6), HFD (n=6), HFD + 2.5 mg/kg Fortunellin (n=6), HFD + 5 mg/kg Fortunellin (n=6), and HFD + 10 mg/kg Fortunellin (n=6). The control group was fed a normal diet for 49 days, and the other groups were fed HFD as the formula in the previous literature [12, 13]. Meanwhile, doses of 2.5, 5, and 10 mg/kg Fortunellin (once a day for 7 weeks) were administered intragastrically to rats a week after the rat NFALD model was established. But control and HFD groups were given an equivalent volume of normal saline [14]. Meanwhile, 0.2 mg/kg compound C (CC, AMPK inhibitor) was administered intragastrically to rats in the HFD+10 mg/kg Fortunellin group [15]. Eventually, rats were euthanized by cervical dislocation and liver tissue was isolated for the next studies. A liver index was calculated by liver tissue weight/rat body weight.

### 2.2 Hematoxylin-eosin (HE) Staining

Liver tissue morphology was examined using HE staining. Following overnight fixation in paraformaldehyde solution at a concentration of 4% (Beyotime, Shanghai, China), liver tissues were sectioned into slices measuring approximately six micrometers thick. Slices were dyed with 5% hematoxylin solution (Beyotime). After washing in distilled water, slices were further exposed to 0.1% hydrochloric alcohol (Beyotime) for 30 s. The morphological changes were obtained with a microscope (Nikon, Japan). The NAFLD Activity Score was conducted based on the previously reported standards [16].

### 2.3 Detection of ALT, AST, TCH, TG, LDL-C, and HDL-C Levels

ALT and AST contents in rat serum samples were checked with standard enzymatic procedures according to the manufacturer's instructions (Jiancheng, Nanjing, China).

TCH (Biovision, Milpitas, USA), TG (Biovision), and HDL-C (Biovision) contents were tested with commercial kits. LDL-C contents were tested using the Friedewald equation as previously reported methodology [17].

### 2.4 Oil Red O Staining

A 6 μm slice of rat liver tissue was dewaxed and rehydrated in xylene. After washing, slices were exposed to Oil Red O reagent (MedChemExpress, Shanghai, China). Next, slices were stained with hematoxylin (Beyotime). After additional washing and dehydration, an Oil Red O and hematoxylin-stained slice was gathered using a fluorescence microscope (Nikon).

### 2.5 Western Blot

Total proteins were extracted from liver tissues *via* RIPA (Beyotime), and protein contents were measured with BCA Protein Assay Kits (Mlbio, Shanghai, China). Then proteins were separated with SDS-PAGE (10%, Sigma-Aldrich, Shanghai, China) and transferred onto PVDF membranes (Millipore, Shanghai, China). Next, membranes were mixed with antibodies against SREBF1 (ab28481, 1/500), PPARγ (ab178860, 1/1000), FABP1 (ab153924, 1/2000), CPT1α (ab220789, 1/1000), LXR (ab315082, 1/1000), FASN (ab128870, 1/10000), IL-1β (ab315084, 1/1000), IL-6 (ab233706, 1/1000), TNF-α (ab183218, 1/1000), p-AMPK (ab92701, 1/2000), AMPK (ab32047, 1/2000), p-ACC (ab222774, 1/1000), ACC (ab109368, 1/5000), and β-actin (ab8226, 1 µg/ml) overnight. Membranes were further exposed to specific secondary antibodies (1/2000) for 2 h. Antibodies were supplied by Abcam (Shanghai, China). Bands were developed *via* ECL kits (Beyotime), and images were assessed with Image J (NIH, Bethesda, USA).

### 2.6 Enzyme-linked Immunosorbent Assay (ELISA)

The serum levels of IL-1β, IL-6, and TNF-β were quantified using the respective ELISA Kits according to the manufacturer's instructions. Quantification was conducted at OD450 values using a microplate reader (ThermoFisher Scientific, Shanghai, China).

### 2.7 Quantitative Real-Time PCR (qRT-PCR)

Total RNA was extracted from the rat liver tissues using TRIzol reagent (ThermoFisher Scientific). Then, cDNA was synthesized by reverse transcription from 1 µg of total RNA with reverse transcription kits (ThermoFisher Scientific). Real-time PCR analysis was performed on the Applied Biosystems 7900 Real-Time PCR system (Applied Biosystems, USA) using the SYBR (ThermoFisher Scientific) and specific primers. Relative quantification of mRNA levels was calculated by the 2^−ΔΔCt^ method and normalized to GAPDH. Primer sequence is as follows: MCP1 forward primer (5'-3'): CAGCCAGATGCAATCAATGCC; reverse primer (5'-3'): TGGAATCCTGAACCCACTTCT; GAPDH forward primer (5'-3'): GGAGCGAGATCCCTCCAAAAT; reverse primer (5'-3'): GGCTGTTGTCATACTTCTCATGG.

### 2.8 Immunofluorescence Analysis

Slices (4 μm) were soaked in 0.3% Triton (Beyotime) for 10 min. Then, slices were blocked by 10% FBS (Beyotime) and incubated overnight with anti-p-AMPK (ab92701, 1/200). After washing, slices were exposed to the corresponding secondary antibody and were further incubated with DAPI (Beyotime). All images were captured using an immunofluorescence microscope (Nikon).

### 2.9 Statistical Analysis

Statistical analyses were executed with GraphPad Prism (version 6.0). Three or more groups’ data were examined using ANOVA followed by Tukey's post hoc test. The two groups’ data were compared *via* Student’s t-test. Data were listed as the mean ± SD. *P* < 0.05 was considered significantly different.

## 3 RESULTS

### 3.1 Fortunellin Alleviates Liver Injury in Young NAFLD Rats

**Fig. (1):**
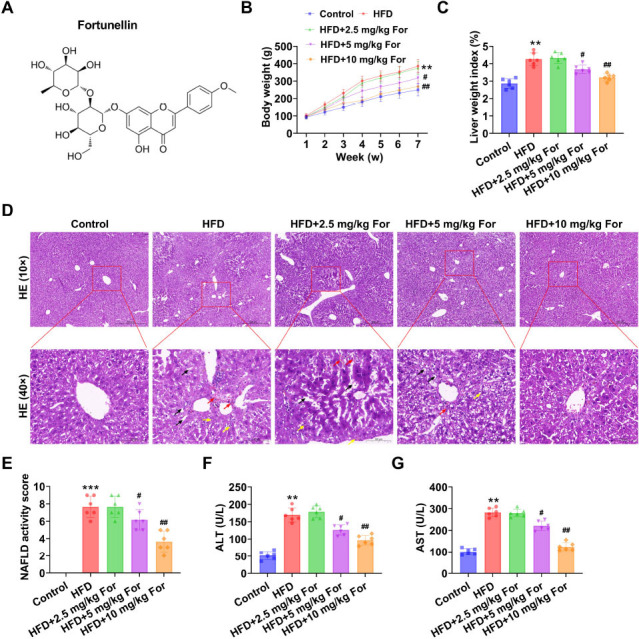
Fortunellin mediates liver injury in young non-alcoholic fatty liver disease (NAFLD) rats. Rats were: control, HFD, HFD + 2.5 mg/kg Fortunellin, HFD + 5 mg/kg Fortunellin, and HFD + 10 mg/kg Fortunellin. (**A**) Molecular formula of Fortunella. (B) Contrast of body weight. (**C**) Analysis of rat liver weight index. (**D-E**) Pathological change of rat liver tissue was validated *via* hematoxylin-eosin (HE) staining. The black arrow represents fat deposition, and the yellow arrow indicates nuclear constriction in liver cells. The red arrow indicates inflammatory cell infiltration. (**F-G**) ALT and AST levels were tested with kits. **P<0.01, ***P<0.001 *vs.* control. ^#^*P*<0.05, ^##^*P*<0.01 *vs.* HFD. HFD: high-fat diet. For: Fortunella.

Fortunellin is a citrus flavonoid extracted from *Fortunella margarita* fruit [8], and its molecular formula is displayed in Fig. (**1A**). This research aimed to investigate the effects of Fortunellin on NAFLD. As shown in Fig. (**1B**), HFD increased the rat's body weight, and administration of Fortunellin at doses of 5 or 10 mg/kg led to a reduction in body weight. Liver weight index of rats in HFD was higher than that in control, yet rat liver index was decreased after Fortunellin (5 or 10 mg/kg) treatment (Fig. **1C**). HE staining further demonstrated that Fortunellin (5 or 10 mg/kg) administration improved HFD-induced liver injury in rats by reducing oxidative stress and mitochondrial injury (Figs. **1D**-**E**). Also, the black arrow represented fat deposition, and the yellow arrow indicated nuclear constriction in liver cells. The red arrow indicated inflammatory cell infiltration (Figs. **1D**-**E**). Moreover, ALT and AST contents in rat serum were raised in HFD, yet their levels were lessened after Fortunellin (5 or 10 mg/kg) administration (Figs. **1F**-**1G**). These data validated that Fortunellin had the function of relieving liver injury in young NAFLD rats.

### 3.2 Fortunellin Improves Lipid Disorders in Young NAFLD Rats

NAFLD refers to a disease characterized by excessive lipid deposition in the liver, along with obesity and metabolic disorders [18]. TCH, TG, LDL-C, and HDL-C are important indicators to determine blood lipid levels [19]. As shown in Figs. (**2A****-****2D**), HFD increased TCH, TG, LDL-C serum contents and reduced HDL-C levels in rats, while these alterations were reversed following treatment with Fortunellin at doses of either 5 or 10 mg/kg. In general, Fortunellin reduced lipid disorders in young NAFLD rats.

### 3.3 Fortunellin Represses Liver Lipid Metabolism in Young NAFLD Rats

**Fig. (3):**
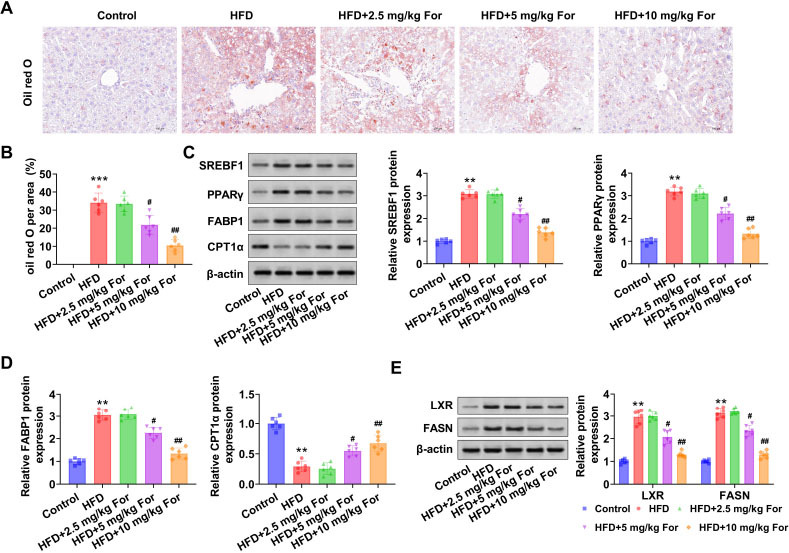
Fortunellin mediates liver lipid metabolism in young NAFLD rats. (**A-B**) Rat liver lipid metabolism was assessed with Oil Red O staining. (**C-D**) Fatty acid synthesis (SREBF1 and PPARγ), fatty acid uptake (FABP1), and fatty acid β-oxidation (CPT1α) protein levels were tested using Western blot. (**E**) The protein levels of lipid metabolism indicators (LXR and FASN) were examined with Western blot. **P<0.01, ***P<0.001 *vs.* control. ^#^*P*<0.05, ^##^*P*<0.01 *vs.* HFD.

Liver lipid metabolism dysfunction is closely associated with NAFLD malignant development [20]. This research further elucidated whether Fortunellin participates in the regulation of liver lipid metabolism. Oil Red O staining data revealed that HFD induced an increase in the percentage of Oil Red O staining in liver tissues, while Oil Red O staining percentage was decreased after Fortunellin (5 or 10 mg/kg) administration (Figs. **3A**-**3B**). SREBF1, PPARγ, FABP1 and CPT1α indicate liver lipid metabolism degree [21, 22]. As shown in (Figs. **3C** and **D**), SREBF1, PPARγ, and FABP1 protein levels in liver tissues were raised in HFD, and CPTα protein levels were lessened, but these alterations were reversed following treatment with Fortunellin. Meanwhile, our experimental data revealed that the protein levels of lipid metabolism indicators (LXR and FASN) were elevated in the HFD group, but this increase was reversed after Fortunellin (5 or 10 mg/kg) treatment (Fig. **3E**). The above experimental data proved that Fortunellin reduced liver lipid metabolism in young NAFLD rats.

### 3.4 Fortunellin Alleviates Immune Disorders in Young NAFLD Rats

**Fig. (4):**
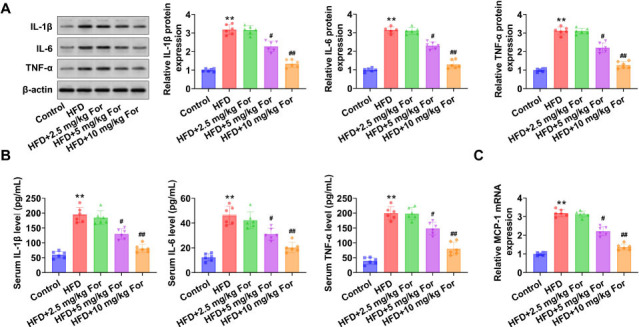
Validation of Fortunellin's regulatory effect on immune disorders in young NAFLD rats. (**A**) L-1β, IL-6, and TNF-α protein levels were determined with Western blot. (**B**) IL-1β, IL-6, and TNF-α levels were tested *via* ELISA. (**C**) The expressions of the inflammatory factor MCPI were measured using quantitative real-time PCR (qRT-PCR). **P<0.01 *vs.* control. ^#^*P*<0.05, ^##^*P*<0.01 *vs.* HFD.

Considering that the immune system exerts an indispensable role in NAFLD [23], we further elucidated the Fortunellin regulation in the immune system in NAFLD. Western blot indicated that HFD induced the increased pro-inflammatory cytokines IL-1β, IL-6, and TNF-α protein levels in liver tissues, yet these alterations were reversed following treatment with Fortunellin at doses of either 5 or 10 mg/kg (Fig. **4A**). Meanwhile, ELISA detection of IL-1β, IL-6, and TNF-α contents in rat serum showed similar trends (Fig. **4B**). Also, the expressions of the inflammatory factor MCPI were increased in the HFD group, but this increase was reversed after Fortunellin (5 or 10 mg/kg) treatment (Fig. **4C**). In short, Fortunellin restrained immune disorders in young NAFLD rats.

### 3.5 Fortunellin Activates the AMPK Pathway in Young NAFLD Rats

**Fig. (5):**
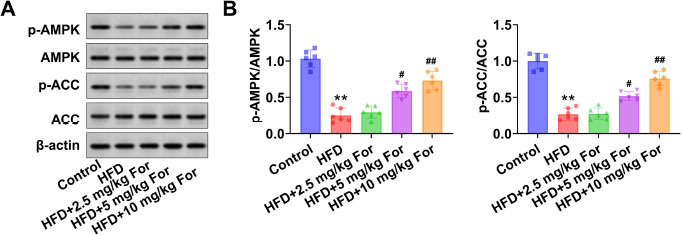
Fortunellin regulates the AMPK pathway in young NAFLD rats. (A-B) Western blot detection of p-AMPK, AMPK, p-ACC, and ACC protein levels in rat liver tissues. **P<0.01 *vs.* control. ^#^*P*<0.05, ^##^*P*<0.01 *vs.* HFD.

Subsequently, we made a preliminary study on the potential mechanism of Fortunellin in alleviating young NAFLD rats. Our data suggested that p-AMPK and p-ACC protein levels were reduced in HFD, while these alterations were reversed following treatment with Fortunellin at doses of either 5 or 10 mg/kg (Figs. **5A** and **B**). These findings hinted that Fortunellin enhanced AMPK activation in young NAFLD rats.

### 3.6 Fortunellin Represses Liver Lipid Metabolism in Young NAFLD Rats by Activating AMPK

**Fig. (6):**
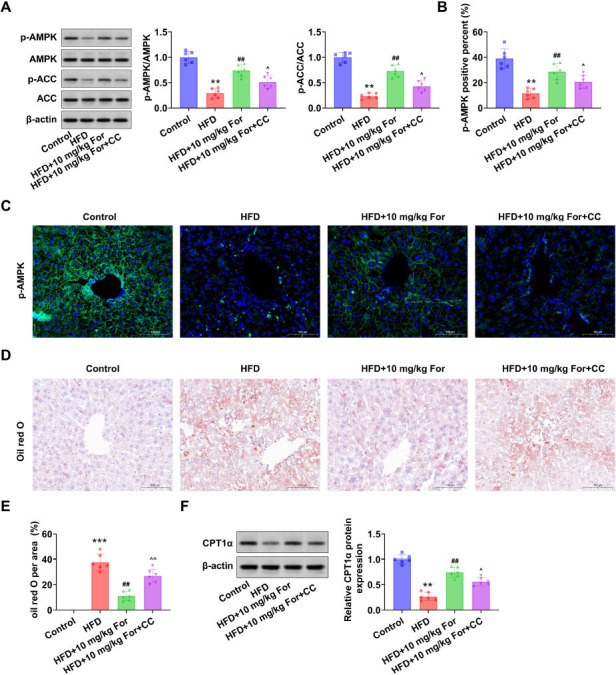
Fortunellin mediates liver lipid metabolism in young NAFLD rats through AMPK. Rats were divided into control, HFD, HFD + 10 mg/kg Fortunellin, and HFD + 10 mg/kg Fortunellin+CC groups. (**A**) Contrast of p-AMPK, AMPK, p-ACC, and ACC protein levels in rat liver tissues using Western blot. (**B-C**) P-AMPK expressions in rat liver tissues were tested with an immunofluorescence assay. (**D-E**) Oil Red O staining analysis of liver lipid metabolism. (**F**) CPT1α protein levels were examined with Western blot. **P<0.01, ***P<0.001 *vs.* control. ^##^*P*<0.01 *vs.* HFD. ^^^*P*<0.05, ^^^^*P*<0.01 *vs.* HFD+10 mg/kg For. CC: compound C.

Our data further demonstrated that Fortunellin increased p-AMPK and p-ACC protein levels in liver tissue, while compound C (CC, AMPK inhibitor) reversed this increase (Fig. **6A**). Immunofluorescence detection of p-AMPK expression in rat liver tissues displayed an analogous trend (Figs. **6B** and **C**). Moreover, Oil Red O staining percentage in rat liver tissues was decreased after Fortunellin treatment, and administration of CC abolished this impact (Figs. **6D** and **E**). Additionally, Fortunellin induced increased CPT1α protein levels in rat liver tissues, yet this increase was abolished after co-treatment with CC (Fig. **6F**). These data implied that Fortunellin relieved liver lipid metabolism in young NAFLD rats *via* activating AMPK.

### 3.7 Fortunellin Reduces Immune Disorders in Young NAFLD rats by Activating AMPK

**Fig. (7):**
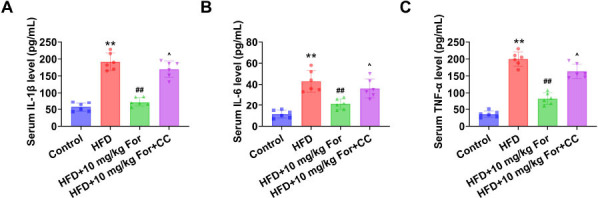
Fortunellin affects immune disorders in young NAFLD rats *via* the AMPK pathway. (A-C) IL-1β, IL-6, and TNF-α levels in rat serum were assessed *via* ELISA. **P<0.01 *vs.* control. ^##^*P*<0.01 *vs.* HFD. ^^^*P*<0.05 *vs.* HFD+10 mg/kg For.

Next, our study data showed that IL-1β, IL-6, and TNF-α contents in rat serum were lessened after Fortunellin treatment, while this impact was abolished after administration of CC (Figs. **7A**-**7C**). In summary, Fortunellin attenuated immune disorders in young NAFLD rats *via* activating AMPK. All in all, Fortunellin reduced lipid deposition and immune disorders in young NAFLD rats through activating AMPK axis.

## 4 DISCUSSION

NAFLD is a global metabolic disease with a high prevalence in children [24, 25]. Considering the protective effect of Fortunellin in multiple human diseases [11, 14], this research tried to elucidate the potential function and mechanism of Fortunellin in NAFLD. The main findings of this research are as follows: (1) Fortunellin reduced liver injury, lipid disorders, liver lipid metabolism, and immune disorders in young NAFLD rats; (2) Fortunellin boosted AMPK activation in young NAFLD rats; (3) Fortunellin weakened lipid deposition and immune disorders in young NAFLD rats through the activation of AMPK. The completion of this research might provide effective drugs for the therapy of NAFLD.

Accumulating studies suggest that the degree of liver injury indicates the construction of an NAFLD animal model. For example, Shi *et al*. proved that Zexie-Baizhu Decoction effectively relieves liver injury induced by NAFLD [26]. Zhu *et al*. validated that Salvianolic acid A alleviates liver injury in NAFLD by the activation of AMPK [27]. Correspondingly, our data stated that Fortunellin reduced NAFLD activity scores, implying that Fortunellin repressed liver injury in young NAFLD rats. Additionally, the abnormally elevated ALT and AST levels are associated with inflammation or injury to the liver, especially in NAFLD [28, 29]. As expected, our experimental data clarified that HFD increased serum ALT and AST levels in rats, whereas Fortunellin treatment reduced these levels, which implied that Fortunellin has the potential to relieve NFALD.

As far as we know, NAFLD refers to a disease characterized by excessive lipid deposition in the liver, along with metabolic disorders such as lipid disorders [30]. A large amount of epidemiological evidence proves that abnormal contents of TCH, TG, LDL-C, and HDL-C are associated with the development of NAFLD. For example, amides in *Z. bungeanum* reduce serum levels of TG, TC, and LDL-C, and raise serum levels of HDL-C in HFD *via* the activation of AMPK/Nrf2, thereby improving NAFLD [31]. Nicotinate-curcumin is effective in NAFLD therapy through decreasing the expressions of LDL-C [32]. After treatment with *Lactobacillus plantarum* Ln4, the total TG levels in the mice's plasma are reduced [33]. Similar to these findings, we confirmed that TCH, TG, and LDL-C levels increased in HFD, and although HDL-C levels decreased, Fortunellin reversed these trends.

More and more evidence reveals that liver lipid metabolism is a significant pathological change of NFALD [34]. As previously reported, Xiaozhi formula is a Chinese herbal medicine that mediates lipid metabolism by the activation of AMPK and PPAR, thereby alleviating NAFLD [35]. Another study also demonstrates that Berberine alleviates NAFLD by targeting the AkR1B10-mediated PPAR pathway to restrain liver lipid metabolism [36]. Crucially, we also validated that Fortunellin reduced the percentage of Oil Red O staining in HFD rat liver tissues. On this basis, we further proved that fatty acid synthesis (SREBF1, PPARγ) and fatty acid uptake (FABP1) protein levels in rat liver tissues were decreased, while fatty acid β-oxidation (CPT1α) was increased after the treatment of Fortunellin. Immune disorder is another pathological factor of NFALD, and abnormal proinflammatory cytokine release is detrimental to the maintenance of immune balance. For instance, N,N'-diacetylcystine reduces the production of IL-6, TNF-α, and IL-1β by down-regulating NF-κB, suggesting that N,N'-diacetylcystine might protect liver injury in HFD-induced NAFLD rats [37]. Artesunate reduces inflammatory factor release in NAFLD through reducing IL-6, IL-1β, and TNF-α levels, supplying a novel approach for NAFLD therapy [38]. Additionally, Fortunellin restrains proinflammatory cytokine secretion and improves the inflammatory response in sepsis-induced AKI [10]. Critically, we demonstrated that Fortunellin decreased IL-1β, IL-6, and TNF-α levels in young NAFLD rat tissues and serum. Our study further suggested that Fortunellin might be an effective drug for the therapy of NAFLD. Subsequently, we would focus on elucidating the potential mechanisms of Fortunellin in mitigating NAFLD.

Energy balance is essential for maintaining body homeostasis [39]. The accompanying imbalance of energy metabolism often results in the development of NAFLD [40]. AMPK is activated to regulate various signaling and metabolic axes to sustain energy homeostasis [41]. In short term, studies have proved that the AMPK axis takes part in mediating the process of NAFLD. For example, Quercetin prevents NAFLD through AMPK-mediated mitochondrial autophagy, suggesting that Quercetin is a promising therapeutic strategy for NAFLD [42]. Rutin activates the AMPK pathway to relieve NAFLD, which suggests that Rutin is a potential drug to treat NAFLD [43]. Meanwhile, Fortunellin protects against inflammation and oxidative stress *via* AMPK in fructose-treated diabetic mice [8]. In this research, we also demonstrated that Fortunellin activated the AMPK axis in young NAFLD rats, and Fortunellin repressed liver lipid metabolism and immune disorders in young NAFLD rats through the activation of AMPK.

## CONCLUSION

To sum up, we offered a novel theoretical basis for Fortunellin-inhibited liver lipid metabolism and immune disorders in young NAFLD rats *via* the activation of AMPK. Our data might suggest Fortunellin as a novel drug for NAFLD therapy. Moreover, this research had the following limitations: (1) No *in vitro* model of NAFLD was explored. (2) Immune-related signaling pathways, such as NF-κB, were not elucidated.

## Figures and Tables

**Fig. (2):**
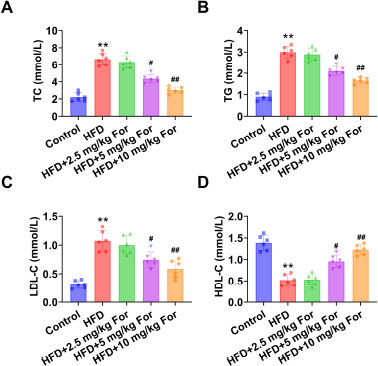
Regulation of Fortunellin on lipid disorders in young NAFLD rats. (**A-D**) TCH, TG, LDL-C, and HDL-C levels were checked with kits. **P<0.01 *vs.* control. ^#^*P*<0.05, ^##^*P*<0.01 *vs.* HFD.

## Data Availability

The datasets used and/or analyzed during the current study are available from the corresponding author on reasonable request.

## LIST OF ABBREVIATIONS

NAFLD: Non-Alcoholic Fatty Liver Disease
HFD: High-Fat Diet
AKI: Acute Kidney Injury
SD: Sprague-Dawley
CC: Compound C
HE: Hematoxylin-eosin
ELISA: Enzyme-linked Immunosorbent Assay
